# A Multi-Mode Switching Variational Bayesian Adaptive Kalman Filter Algorithm for the SINS/PNS/GMNS Navigation System of Pelagic Ships

**DOI:** 10.3390/s22093372

**Published:** 2022-04-28

**Authors:** Jie Zhang, Shanpeng Wang, Wenshuo Li, Zhenbing Qiu

**Affiliations:** 1 School of Automation Science and Electrical Engineering, Beihang University, Beijing 100191, China; zhangjie_buaa@buaa.edu.cn; 2 School of Instrumentation and Optoelectronic Engineering, Beihang University, Beijing 100191, China; spwangbuaa@126.com; 3 Hangzhou Innovation Institute, Beihang University, Hangzhou 310051, China

**Keywords:** multi-mode switching, variational Bayesian, adaptive filter, autonomous navigation

## Abstract

The ocean-going environment is complex and changeable with great uncertainty, which poses a huge challenge to the navigation ability of ships working in the pelagic ocean. In this paper, in an attempt to deal with the complex uncertain interference that the environment may bring to the strap-down inertial navigation system/polarization navigation system/geomagnetic navigation system (SINS/PNS/GMNS) integrated navigation system, the multi-mode switching variational Bayesian adaptive Kalman filter (MMS-VBAKF) algorithm is proposed. To be more specific, to identify the degrees of measurement interference more effectively, we design an interference evaluation and multi-mode switching mechanism using the original polarization information and geomagnetic information. Through this mechanism, the interference to the SINS/PNS/GMNS navigation system is divided into three cases. In case of slight interference (case SI), the variational Bayesian method is adopted directly to solve the problem that the statistical characteristics of measurement noise are unknown. By the fixed-point iteration mechanism, the statistical properties of the measurement noise and the system states can be estimated adaptively in real time. In case of interference-tolerance (case TI), the estimation of the statistical characteristics of measurement noise need to weigh the measurement information at the moment and a priori value information comprehensively. In case of excessive interference (case EI), the SINS/PNS/GMNS integrated navigation system will perform mode switching and filtering system reconstruction in advance. Then, the information fusion and navigation states estimation can be completed. Consequently, the reliability, robustness, and accuracy of the SINS/PNS/GMNS integrated navigation system can be guaranteed. Finally, the effectiveness of the algorithm is illustrated by the simulation experiments.

## 1. Introduction

With the advancement of activities such as marine energy resource exploration, maritime trade and transportation, and marine biodiversity research, far-sea missions have become increasingly frequent. Reliable navigation is one of the key technologies to ensure the smooth completion of the far-sea tasks. In the offshore field, commonly used means for navigation include inertial navigation [1], acoustic navigation [2], geophysical field matching navigation [3], satellite navigation [4], and visual navigation [5]. When a ship is working in an unfamiliar pelagic environment, however, the navigation methods it can rely on are extremely limited. In fact, it is quite difficult to build accurate geophysical databases (such as terrain databases and altitude databases) in unfamiliar far-sea areas, and there is no complete underwater acoustic base station to rely on. Furthermore, the unstructured property and the uncertain reflection effect of the sea surface will also lead to failure of visual navigation. Inertial navigation, despite its capability to provide uninterrupted navigation information all day and in all weather, has an inherent deficiency that the navigation error will accumulate with time. Large errors will be produced if inertial navigation is used alone for long-term tasks. Due to its high precision, the satellite navigation can serve as a suitable option to correct inertial navigation errors. However, the power of satellite navigation signal is low, which makes it quite sensitive to either intentional or unintentional interferences [6]. Hence, it is urgent to develop an autonomous navigation method that is available under the condition that satellite navigation is out of work.

In order to overcome the aforementioned shortcomings of the existing navigation schemes and achieve reliable navigation in the far-sea environments, a fully autonomous integrated navigation scheme based on SINS/PNS/GMNS will be developed in this paper. As an emerging method for autonomous navigation, polarization navigation extracts navigation information from the polarization pattern of the skylight [7,8,9,10]. It has been found in the existing studies that a variety of animals (such as ants [11], bees [12], monarch butterflies [13], crickets [14], etc.) can perceive the polarized light distribution pattern in the sky to distinguish the direction, thereby guiding their foraging, homing, and other behaviors. As a traditional geophysical navigation method, geomagnetic navigation is not affected by time or spatial factors and can be used as an effective complement of inertial navigation when the polarization sensors become unreliable overnight [15].

To make the best performance of the SINS/PNS/GMNS integrated navigation system, it is necessary to have a comprehensive understanding of system noise. When using geomagnetic information for navigation, both hard and soft magnetic interferences caused by the carrier steel structure and electrical module should be considered [16,17,18,19]. On the other hand, the skylight polarization mode is affected by complex atmospheric conditions [20,21,22,23,24]. Specifically, under cloudy or hazy weather conditions, the degree of polarization (DoP) drops sharply and the angle of polarization (AoP) would also be distorted. Raymond et al. [23] collected the polarization data of clear sky and foggy sky in the seaside area, compared it with the polarization spectrum of aerosol single scattering, and illustrated the difference of polarization in different weather. Ma et al. [24] conducted a quantitative analysis of the difference of AoPs under different weather conditions and investigated the relationship between DoP and AoP distortion. Moreover, due to the polarization sensing mechanism, the data obtained from the polarization sensors are affected by noises with complicated characteristics [25,26,27,28]. Up to date, many elegant results on noise treatment have been reported. Chu et al. [29] constructed a compact on-board polarization orientation system and proposed an adaptive robust algorithm based on information entropy theory. Zhao et al. [30] proposed a self-learning multi-rate residual calibration algorithm for the INS/polarization integrated system to deal with the system noise. Shen et al. [31] designed an INS/GPS/polarization compass system and proposed the MR-STSCKF algorithm to deal with noisy observation as well as the problem of inconsistent sampling frequency. To improve the accuracy of the polarization/geomagnetic/GNSS/MIMU integrated navigation system under environmental interferences, Yuan et al. [32] proposed an adaptive filtering algorithm based on Sage–Husa Kalman filter. It should be noted, however, that the aforementioned approaches share an implicit assumption that the measurement noises are Gaussian random variables, which is too restrictive for practical navigation systems operating under harsh environments.

Ships may encounter harsh conditions in the unfamiliar pelagic environment, such as cloudy and rainy weather which is harsh on the polarization sensor. Under such conditions, the sky atmospheric polarization distribution pattern might be destroyed or completely depolarized, and the polarization navigation system will fail to work. Moreover, once the ship enters an iron–nickel mining sea area, the geomagnetic measurement data will be completely distorted. Therefore, it is essential to set up an interference detection and evaluation mechanism for the SINS/PNS/GMNS integrated navigation system. Reliable navigation has become a research hotspot [33,34,35]. Zhang et al. [33] adopted the method of manually setting the evaluation value and federated filtering to conduct interference processing. To cope with the model errors and uncertain interference, Chen et al. [34] proposed a method that integrates the advantages of the H-infinity filter and multiple fading filter and improves the robustness of the navigation system. Even though a certain degree of success has been achieved with the existing methods, how to cope with the complex interference in the INS/PNS/GMNS integrated navigation system still remains an unsolved issue.

In this paper, we propose a multi-mode switching variational Bayesian adaptive Kalman filtering approach to ensure both accuracy and reliability of the SINS/PNS/GMNS integrated navigation system. In order for a more accurate noise description, the noises in the PNS/GMNS measurement model are treated as inverse Wishart distribution statistics characteristics. On this basis, the variational Bayesian filtering algorithm is adopted for joint estimation of the measurement noise covariance, the system state, and the state error covariance. Furthermore, to ensure the stability of real-time filtering under abnormal state, an interference evaluation and mode switching mechanism will be developed based on the original polarization and geomagnetic data. With the proposed scheme, a more accurate state estimate can be obtained when the system operates normally and reliable navigation can be guaranteed when harsh interference occurs.

In summary, for the autonomous navigation requirements of ships in unfamiliar pelagic scenarios, the technical advantages of the proposed scheme can be summarized as follows:

- As an autonomous integrated system for ocean-going ships, the SINS/PNS/GMNS integrated navigation system is subject to complex environmental interference. Hence, the statistics of the measurement noises should be estimated in real time instead of being prescribed as constant. In this paper, the variational Bayesian method is adopted to estimate the noise statistics along with the system state based on real-time observations from PNS and GMNS.
- The reliability of the SINS/PNS/GMNS integrated navigation system is affected by the uncertainties in pelagic environment. For example, severe degradation of the accuracy in the integrated navigation system might be caused when the ship encounters harsh weather conditions or travels through an iron–nickel mining sea area. In order to enhance the reliability of the SINS/PNS/GMNS integrated navigation system, an interference evaluation algorithm and mode switching mechanism are developed in this paper. With the proposed scheme, the abrupt change resulting from harsh environmental conditions can be detected timely and the filtering mode can be switched smoothly, thereby ensuring the reliability of the integrated navigation system.

The paper is organized as follows. In Section 2, the system state model and measurement model of the SINS/PNS/GMNS integrated navigation system are established at first. Next, to deal with the unknown time-varying noise statistics in the PNS/GMNS observations, the VBAKF algorithm is introduced. Furthermore, for the sake of the possible uncertainty in the pelagic sea scenario, the interference evaluation mechanism and multi-mode switching algorithm are designed to improve system reliability. Lastly, the MMS-VBAKF algorithm is summarized. In Section 3, the performance of the proposed algorithm is verified by simulation experiments in three scenarios. Finally, a brief conclusion is given in Section 4.

## 2. Methods

### 2.1. SINS/PNS/GMNS Integrated Navigation Model and the Conjugate Prior Distribution of Noise

#### 2.1.1. SINS/PNS/GMNS Integrated Navigation Model

**Figure 1:**
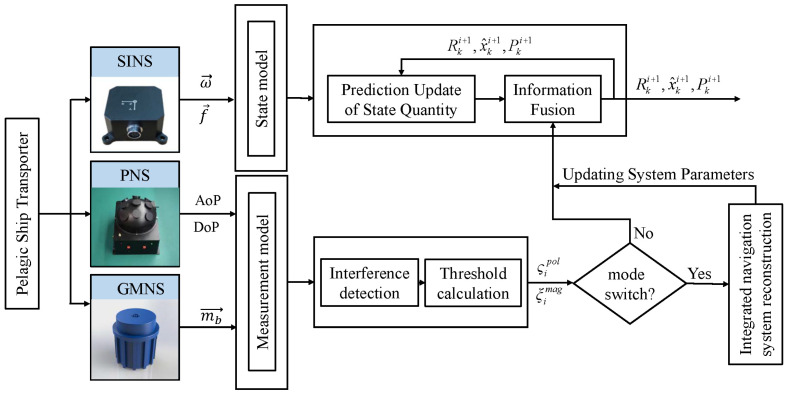
The SINS/PNS/GMNS integrated navigation filter architecture diagram.

For the integrated navigation system of ocean-going ships, the centralized filtering is the main form adopted by the current maritime ship system. Therefore, in this paper, we also adopt centralized filtering to realize SINS/PNS/GMNS integrated navigation, and its basic framework is shown in Figure 1.

In this design system, the integrated navigation system state model is

(1) $$\dot{x}=\Phi \cdot x+G\cdot w$$

where $x={\left[\begin{array}{ccccccccc} \phi_{E} & \phi_{N} & \phi_{U} & \varepsilon_{x} & \varepsilon_{y} & \varepsilon_{z} & \nabla_{x} & \nabla_{y} & \nabla_{z} \end{array}\right]}^{T}$ represents the system state variables, in which $\phi_{E},\phi_{N},\phi_{U}$ represents attitude angle errors of east, north, and zenith; $\varepsilon_{x},\varepsilon_{y},\varepsilon_{z}$ denotes the gyro drift errors; $\nabla_{x},\nabla_{y},\nabla_{z}$ denotes the three-axis accelerometer bias errors; w denotes the system excitation white noise; Φ denotes the state transition matrix; G denotes the process noise driving array. Φ and G can be determined by the strap-down inertial navigation errors equation [36].

The measurement information of the integrated navigation system consists of polarization measurement and geomagnetic measurement. First of all, for the polarization system, the measurement model is established based on the formation mechanism of polarization distribution patterns, that is, the vertical relationship between the sun vector and the polarization vector.

(2) $$s_{n}\cdot {\tilde{C}}_{b}^{n}\left(1+\Delta \psi \right)p_{b}=0$$

where $s_{n}$ is the solar vector which can be obtained by querying the astronomical almanac according to the time and position information of the carrier; ${\tilde{C}}_{b}^{n}$ is the attitude transformation matrix from the machine system to the navigation system solved by the navigation system; Δψ is the error angle between the calculated three-dimensional attitude and the real three-dimensional attitude; $p_{b}$ is the polarization vector measured by the polarization sensor in real time. Equation (2) can be further arranged into the measurement equation:

(3) $$z_{pol}=H_{pol}x+v_{pol}$$

where $z_{pol}$ is the polarization measurement; $H_{pol}$ is the polarization measurement matrix, specifically expressed as $H_{pol}=\left[s_{n}\cdot {\left({\tilde{C}}_{b}^{n}p_{b}\right)}^{\times },0_{1\times 6}\right]$, ${\left(\cdot \right)}^{\times }$ is the antisymmetric operator; $v_{pol}$ is the polarization measurement noise with unknown and time-varying statistical information.

Next, in the geomagnetic measurement model, considering the one-to-one correspondence between the geomagnetic vector output by the world magnetic model (WMM) and that measured by the geomagnetic sensor in the same geographical location and time, the geomagnetic measurement model is established as follows:

(4) $$z_{mag}={\tilde{C}}_{b}^{n}m_{b}-m_{n}$$

where $z_{mag}$ is the geomagnetic measurement; $m_{n}$ is a three-dimensional geomagnetic measurement given by WMM; and $m_{b}$ is a three-dimensional magnetic vector measured by the magnetic sensor. Equation (4) can be further sorted out, giving

(5) $$z_{mag}=H_{mag}x+v_{mag}$$

where $H_{mag}$ is the geomagnetic measurement matrix, specifically expressed as $H_{mag}=\left[{\left(C_{b}^{n}M_{b}\right)}^{\times },0_{3\times 6}\right]$; $v_{mag}$ is geomagnetic measurement noise, and in this paper is unknown time-varying noise.

In summary, the measurement model of the SINS/PNS/GMNS integrated navigation system is established as

(6) $$z=\left[\begin{array}{c} z_{pol}\\ z_{mag} \end{array}\right]=\left[\begin{array}{c} H_{pol}\\ H_{mag} \end{array}\right]\cdot x+\left[\begin{array}{c} v_{pol}\\ v_{mag} \end{array}\right]=Hx+v$$

#### 2.1.2. Conjugate Prior Distribution of Measurement Noise

For the constructed integrated navigation system model, it is assumed that the distribution of one-step state prediction and that of the measurement likelihood both satisfy the normal distribution, i.e.,

(7) $$\left\{ \begin{array}{l} p\left(x_{k}|z_{1:k-1},P_{k|k-1}\right)=N\left(x_{k};{\hat{x}}_{k|k-1},P_{k|k-1}\right)\\ p\left(z_{k}|x_{k},R_{k}\right)=N\left(z_{k};H_{k}x_{k},R_{k}\right) \end{array}\right.$$

where N(⋅) represents a normal distribution; $x_{k},z_{k},R_{k},H_{k}$, respectively represent the state vector, measurement vector, measurement error covariance matrix, and state transition matrix at time k; $z_{1:k-1}$ represents the measurement from time 1 to k−1; ${\hat{x}}_{k|k-1}$ represents the one-step state estimation vector; $P_{k|k-1}$ represents the one-step estimated state error covariance matrix. The calculation can be obtained according to the following formula:

(8) $$\left\{ \begin{array}{l} {\hat{x}}_{k|k-1}=F_{k}{}_{-1}{\hat{x}}_{k-1}\\ P_{k|k-1}=F_{k-1}P_{k-1}F_{k-1}^{T}+Q_{k-1} \end{array}\right.$$

where ${\left(\cdot \right)}^{T}$ represents the transpose operator; $F_{k-1}$ represents the state transition matrix at k−1 after the discretization of the state equation; $Q_{k-1}$ represents the systematic error covariance matrix of state prediction error. In the integrated navigation filtering algorithm, inverse Wishart (IW) distribution is usually selected as the conjugate prior distribution of the error covariance matrix, and the specific function form is [37]

(9) $$IW\left(\Lambda ;\lambda ,\Theta \right)=\frac{{\left|\Theta \right|}^{\frac{\lambda }{2}}{\left|\Lambda \right|}^{\frac{-\left(\lambda +d+1\right)}{2}}\exp\left\{-\frac{1}{2}tr\left(\Theta \Lambda^{-1}\right)\right\}}{2^{\frac{1}{2}d\lambda }\Gamma_{d}\left(\frac{\lambda }{2}\right)}$$

where λ is the degree of freedom parameter. d is the dimension of the matrix. Θ is the inverse scale matrix, which is a d×d—dimensional symmetric positive definite matrix. tr(⋅) is the trace operator. $\Gamma_{d}\left(\cdot \right)$ is the Gamma distribution of the d variable. In this paper, inverse Wishart distribution is selected as conjugate prior distribution of state noise covariance matrix and measurement noise covariance, which can be given as

(10) $$\left\{ \begin{array}{l} p\left(P_{k|k-1}|z_{1:k-1}\right)=IW\left(P_{k|k-1};{\hat{c}}_{k|k-1},{\hat{C}}_{k|k-1}\right)\\ p\left(R_{k}|z_{1:k-1}\right)=IW\left(R_{k};{\hat{u}}_{k|k-1},{\hat{U}}_{k|k-1}\right) \end{array}\right.$$

where ${\hat{c}}_{k|k-1}$ and ${\hat{C}}_{k|k-1}$ are the degree of freedom parameters and inverse scale matrix of state error covariance matrix, respectively. ${\hat{u}}_{k|k-1}$ and ${\hat{U}}_{k|k-1}$ are the degree of freedom parameters and inverse scale matrix of the measurement error covariance matrix, respectively.

For the inverse Wishart distribution, when λ>d+1, the inverse Wishart distribution satisfies $E\left[\Lambda^{-1}\right]=\left(\lambda -d-1\right)\Theta^{-1}$. Thus, the one-step update of each parameter can be deduced [38]:

(11) $$\left\{ \begin{array}{l} {\hat{c}}_{k|k-1}=n+\tau +1\\ {\hat{C}}_{k|k-1}=\tau P_{k|k-1}\\ {\hat{u}}_{k|k-1}=\rho \left({\hat{u}}_{k-1}-m-1\right)+m+1\\ {\hat{U}}_{k|k-1}=\rho {\hat{U}}_{k-1} \end{array}\right.$$

where τ is the non-negative regulating parameter. ρ is the attenuation factor, and it satisfies 0<ρ≤1.

To sum up, this section completes modeling of the SINS/PNS/GMNS integrated navigation system and modeling of the noise prior distribution of the measurement system.

### 2.2. Adaptive Posterior Estimation Based on Variational Bayesian Algorithm

When the measurement information of the SINS/PNS/GMNS integrated navigation system is updated, the probability distribution of filtering estimation parameters can be estimated posteriorly by using real-time polarization information and geomagnetic information, which can improve the real-time calculation accuracy of the integrated navigation system. For the proposed ship integrated navigation system model, the following formula can be obtained according to the Bayesian theory:

(12) $$\begin{array}{ll} p\left(x_{k},P_{k|k-1},R_{k}|z_{1:k}\right) & =\frac{p\left(x_{k},P_{k|k-1},R_{k},z_{k}\right)}{p\left(z_{1:k}\right)}\\ & =\frac{p\left(z_{k}|x_{k},P_{k|k-1},R_{k},z_{1:k-1}\right)p\left(x_{k},P_{k|k-1},R_{k}|z_{1:k-1}\right)}{p\left(z_{k}|z_{1:k-1}\right)} \end{array}$$

The measured value at time *k* is set to be independent of the measured value at any previous time, and the above equation can be written as

(13) $$p\left(x_{k},P_{k|k-1},R_{k}|z_{1:k}\right)=\frac{p\left(z_{k}|x_{k},P_{k|k-1},R_{k}\right)p\left(x_{k},P_{k|k-1},R_{k}|z_{1:k-1}\right)}{p\left(z_{k}\right)}$$

If the maximum posterior probability distribution functions of parameters $\left\{x_{k},P_{k|k-1},R_{k}\right\}$ are estimated directly by Bayesian theory, it is difficult to obtain accurate analytical solutions of $\left\{x_{k},P_{k|k-1},R_{k}\right\}$ due to the coupling relationship among parameters. A new probability distribution function $q\left(x_{k},P_{k|k-1},R_{k}\right)$ of $\left\{x_{k},P_{k|k-1},R_{k}\right\}$ is set to approximate the real posterior probability distribution function $p\left(x_{k},P_{k|k-1},R_{k}|z_{1:k}\right)$ by using the variational method, and the mean-field theory (MFT) is applied to $q\left(x_{k},P_{k|k-1},R_{k}\right)$ [37,38,39]; it can be obtained:

(14) $$q\left(x_{k},P_{k|k-1},R_{k}\right)=q\left(x_{k}\right)\cdot q\left(P_{k|k-1}\right)\cdot q\left(R_{k}\right)$$

To make the probability distribution function $q\left(x_{k},P_{k|k-1},R_{k}\right)$ sufficiently approximate the true posterior probability distribution function $p\left(x_{k},P_{k|k-1},R_{k}|z_{1:k}\right)$, it can be obtained by minimizing the Kullback–Leibler divergence (KLD) of the two probability density functions, i.e.,

(15) $$\begin{array}{l} \left\{q\left(x_{k}\right),q\left(P_{k|k-1}\right),q\left(R_{k}\right)\right\}\\ =\arg\min D_{KL}\left(q\left(x_{k},P_{k|k-1},R_{k}\right)||p\left(x_{k},P_{k|k-1},R_{k}|z_{1:k}\right)\right)\\ =\arg\min D_{KL}\left(q\left(\kappa \right)||p\left(\kappa |z_{1:k}\right)\right) \end{array}$$

where the KLD is defined as $D_{KL}\left(q\left(x\right)||p\left(x\right)\right)≜\int q\left(x\right)\ln\frac{q\left(x\right)}{p\left(x\right)}dx$, and $\kappa =\left\{x_{k},P_{k|k-1},R_{k}\right\}$.

Equation (15) can be solved and simplified to obtain

(16) $$\ln p\left(z_{1:k}\right)=D_{KL}\left(q\left(\kappa \right)||p\left(\kappa |z_{1:k}\right)\right)+\int q\left(\kappa \right)\ln\frac{p\left(\kappa ,z_{1:k}\right)}{q\left(\kappa \right)}d\kappa$$

In Equation (16), since $D_{KL}\left(\cdot \right)\geq 0$, $L\left(q\left(\kappa \right)\right)=\int q\left(\kappa \right)\ln\frac{p\left(\kappa ,z_{1:k}\right)}{q\left(\kappa \right)}d\kappa$ can be the lower bound of $\ln p\left(z_{1:k}\right)$ and is usually referred to as the evidence lower bound (ELOB) of $\ln p\left(z_{1:k}\right)$. Therefore, solving the minimum value of $D_{KL}\left(q\left(\kappa \right)||p\left(\kappa |z_{1:k}\right)\right)$ can be equivalent to finding the maximum ELOB of $\ln p\left(z_{1:k}\right)$, that is, the problem can be transformed into finding the maximum value of L(q(κ)):

(17) $$\begin{array}{l} \arg\max L\left(q\left(\kappa \right)\right)\\ =\arg\max\left\{\int q\left(\kappa \right)\ln p\left(\kappa ,z_{1:k}\right)d\kappa -\int q\left(\kappa \right)\ln q\left(\kappa \right)d\kappa \right\} \end{array}$$

Further, it can be obtained that

(18) $$\ln q\left(\tau \right)=E_{\kappa (-\tau )}\left\{\ln p\left(\kappa ,z_{1:k}\right)\right\}+C_{\tau }$$

where E{⋅} represents the expectation operator, τ represents any parameter in $\left\{x_{k},P_{k|k-1},R_{k}\right\}$, κ(−τ) represents other parameters of $\left\{x_{k},P_{k|k-1},R_{k}\right\}$ excluding τ, and $C_{\tau }$ represents constant values related to τ.

According to the probability distribution model of state quantity, measurement quantity, and noise of PINS/PNS/MNS integrated navigation system, the joint probability density function can be obtained. Further, according to MFT and Equation (14), the fixed-point iteration method is adopted to update $x_{k},P_{k|k-1},R_{k}$ respectively. In consequence, the posterior probability distribution parameter of $x_{k}$ can be updated as

(19) $$\left\{ \begin{array}{l} {\hat{x}}_{k}^{i+1}={\hat{x}}_{k|k-1}+K_{k}^{i+1}\left(z_{k}-H_{k}{\hat{x}}_{k|k-1}\right)\\ P_{k}^{i+1}=P_{k|k-1}^{i+1}-K_{k}^{i+1}H_{k}P_{k|k-1}^{i+1} \end{array}\right.$$

In which, $K_{k}^{i+1}$ satisfies $K_{k}^{i+1}=P_{k|k-1}^{i+1}H_{k}^{T}{\left(H_{k}P_{k|k-1}^{i+1}H_{k}^{T}+R_{k}^{i+1}\right)}^{-1}$.

In conclusion, the variational Bayesian framework is adopted to derive the posterior probability density parameter update of $\left\{x_{k},P_{k|k-1},R_{k}\right\}$ by fixed-point iteration technology. When the measurement data arrive, the variational Bayesian filter can estimate the posterior probability distribution of $\left\{x_{k},P_{k|k-1},R_{k}\right\}$ in real time, and obtain more accurate navigation parameters, which have excellent real-time performance.

### 2.3. Interference Evaluation Algorithm and Mode Switching Mechanism of the SINS/PNS/GMNS Integrated System

In order to improve the operating reliability of the SINS/PNS/GMNS integrated navigation system, the interference evaluation algorithm and multi-mode switching mechanism are designed. For instance, rainy or cloudy weather can cause serious interference to polarization data, and submarine magnetic minerals can cause local magnetic field distortion. On these occasions, the measurement interference should be detected and evaluated quickly, parts of the system will be isolated, and the filter model will be restructured to guarantee the reliability of the ship’s integrated navigation system. In this section, only the interference on polarization navigation system or geomagnetic navigation system is considered.

#### 2.3.1. Interference Evaluation Algorithm and Multi-Mode Switching Mechanism of the Polarization System

The navigation accuracy calculated based on the polarization data depends on whether the atmosphere has an obvious scattering distribution to the sunlight. When the weather is clear, the atmospheric composition is relatively pure, the polarization mode formed by scattering is less affected by large particle suspensions and aerosols, and the obtained polarization data have a high signal-to-noise ratio. On the contrary, the signal-to-noise ratio of the polarization data measured in cloudy or rainy weather is extremely low. The DoP value is the physical quantity that characterizes the proportion of polarized light in the total skylight intensity, which can effectively measure the credibility of polarization data:

(20) $$\mathrm{DoP}=\frac{I_{in}-I_{in}\cdot \cos^{2}\alpha }{I_{in}+I_{in}\cdot \cos^{2}\alpha }=\frac{1-\cos^{2}\alpha }{1+\cos^{2}\alpha }$$

where $I_{in}$ indicates the intensity of incident light, and α indicates the polarization angle measured by the polarization sensor.

There are water molecules, aerosols, and other components in the atmosphere near the sea surface, which will bring uncertain interference to polarization data. In addition, changing weather conditions at sea, such as sudden rain, can even cause polarization sensors to fail. In order to deal with the interference of the polarization system more effectively, the interference evaluation mechanism of the polarization system is designed. With the interference evaluation parameter $\varsigma^{pol}$, it is divided into three cases:

(21) $$\varsigma_{i}^{pol}=\{\begin{array}{c} 1,\\ \frac{DoP_{i}}{1-DoP_{i}},\\ 0, \end{array}\begin{array}{c} Th_{pol}^{u}<DoP_{i}\leq 1\\ Th_{pol}^{d}<DoP_{i}<Th_{pol}^{u}\\ DoP_{i}\leq Th_{pol}^{d} \end{array}$$

In Equation (21), $\varsigma_{i}^{pol}$ indicates the interference evaluation parameter. $Th_{pol}^{u}$ indicates the upper bound threshold; when the $\mathrm{DoP}>Th_{pol}^{u}$, it means that the interference is slight and can be handled smoothly by the system filter. The parameter $Th_{pol}^{u}$ should be determined by the actual accuracy requirements for the navigation task. $Th_{pol}^{d}$ indicates the lower bound threshold; when the $\mathrm{DoP}\leq Th_{pol}^{d}$, it means that the polarization pattern is badly disrupted. The parameter $Th_{pol}^{d}$ should be determined by the performance of the polarization sensor. According to the degree of interference in polarization data, it is discussed as the following three cases:

- Case I: When $\mathrm{DoP}>Th_{pol}^{u}$, the interference evaluation parameter $\varsigma_{i}^{pol}$ satisfies $\varsigma_{i}^{pol}=1$. The physical meaning is that when the polarization sensor works under the condition that DoP value is above the upper bound threshold $Th_{pol}^{u}$, there are slight interference noises in the acquired measurement data. The weight of polarization navigation is equal to 1. The states of the navigation system can be estimated by the VBAKF algorithm smoothly in time. Accordingly, this case is defined as the case of slight interference, i.e., Case SI.
- Case II: When $Th_{pol}^{d}<DoP_{i}<Th_{pol}^{u}$, the interference evaluation parameter $\varsigma_{i}^{pol}$ satisfies $0<\varsigma_{i}^{pol}<1$. It is indicated that the polarization sensor is working with a certain extent interference. In this case, the estimation of noise statistical properties needs to weigh the measurement information and the prior noise information at the same time, and then estimate the measurement noise comprehensively. Accordingly, this case is defined as the case of interference-tolerance, i.e., Case TI. The measurement noise covariance can be estimated as:
  (22) $${\hat{R}}_{k}^{pol}=\left(1-\varsigma_{i}^{pol}\right)R_{k-1}^{pol}+\varsigma_{i}^{pol}{\tilde{R}}_{k}^{pol}$$

In Equation (22), $R_{k-1}^{pol}$ indicates the prior measurement error covariance; ${\tilde{R}}_{k}^{pol}$ indicates the measurement error covariance calculated by the VBAKF algorithm with the measurement data in real-time.

- Case III: When $\mathrm{DoP}\leq Th_{pol}^{d}$, the interference evaluation parameter $\varsigma_{i}^{pol}$ turns out to be $\varsigma_{i}^{pol}=0$. In this case, it indicates that the degree of interference exceeds the handling ability of the polarization system, and the polarization system is judged to be invalid. Accordingly, this case is defined as the case of excessive interference, i.e., Case EI. Further, with $\varsigma_{i}^{pol}=0$, the polarization system will be isolated and the filter should be reconstructed. The system measurement model will switch to be:
  (23) $$z=z_{i}^{mag}=H_{i}^{mag}x_{i}+v_{i}^{mag}$$

#### 2.3.2. Interference Evaluation Algorithm and Mode Switching Mechanism of the Geomagnetic System

When the shipborne magnetic sensor measures geomagnetic data, in one aspect, the navigation accuracy is affected by the hard magnetic interference and soft magnetic interference. For the other, if the ship encounters a submarine magnetic mining area, the abnormal magnetic field can directly lead the measured data to be distorted completely. Therefore, to ensure the credibility of GMNS, the following magnetic interference detection and evaluation mechanism are designed:

(24) γ=2⋅abs(msx2+msy2+msz2−|MnF|)|MnF|

where msx,msy,msz, respectively, represent the components of the geomagnetic vector on the x, y, and z axes in the sensor coordinate system. MnF indicates the total intensity of the geomagnetic vector in the navigation coordinate system. γ represents the interference indication parameter of geomagnetic data.

(25) $$\xi_{i}^{mag}=\{\begin{array}{c} 1,\\ \alpha \cdot \gamma_{i},\\ 0, \end{array}\begin{array}{c} 0\leq \gamma_{i}\leq Th_{mag}^{d}\\ Th_{mag}^{d}<\gamma_{i}\leq Th_{mag}^{u}\\ \gamma_{i}>Th_{mag}^{u} \end{array}$$

In the above formula, $\xi_{i}^{mag}$ indicates the interference evaluation parameter of the geomagnetic system; α indicates the normalization factor. $Th_{mag}^{u}$ indicates the upper bound threshold; when the $\gamma_{i}>Th_{mag}^{u}$, it means that the interference is too much and geomagnetic data are distorted severely. The parameter $Th_{mag}^{u}$ should be determined by the performance of the geomagnetic sensor. $Th_{mag}^{d}$ indicates the lower bound threshold; when the $\gamma_{i}\leq Th_{mag}^{d}$, it means that the interference is slight and can be handled smoothly by the system filter. The parameter $Th_{mag}^{d}$ should be determined by the actual accuracy requirements for the navigation task. According to the degree of interference in geomagnetic data, it is discussed as the following three cases:

- Case I: When $0\leq \gamma_{i}\leq Th_{mag}^{d}$, the interference evaluation parameter $\xi_{i}^{mag}=1$. In this case, the interferences in GMNS data are slight and can be effectively estimated and compensated by the VBAKF algorithm. Accordingly, this case is defined as the case of slight interference, i.e., Case SI.
- Case II: When $Th_{mag}^{d}<\gamma_{i}\leq Th_{mag}^{u}$, the interference evaluation parameter $\xi_{i}^{mag}$ is calculated to be a normalized weight coefficient. In this case, the GMNS should work with a certain extent interference. Thus, the estimation of noise statistics needs to weigh the measurement information and the prior noise information at the same time. Accordingly, this case is defined as the case of interference-tolerance, i.e., Case TI. The measurement noise covariance can be estimated comprehensively as:
  (26) $${\hat{R}}_{k}^{mag}=\left(1-\xi_{i}^{mag}\right)R_{k-1}^{mag}+\xi_{i}^{mag}{\tilde{R}}_{k}^{mag}$$
  In Equation (26), $R_{k-1}^{mag}$ indicates the prior measurement error covariance; ${\tilde{R}}_{k}^{mag}$ indicates the measurement error covariance calculated by the VBAKF algorithm.
- Case III: When $\gamma_{i}>Th_{mag}^{u}$, the interference evaluation parameter $\xi_{i}^{mag}=0$. In this case, it indicates that the Earth’s magnetic field is disturbed severely by abnormal magnetic fields, and the GMNS system cannot work effectively. Accordingly, this case is defined as the case of excessive interference, i.e., Case EI. Further, with $\xi_{i}^{mag}=0$, the geomagnetic system will be isolated and the filter will be reconstructed. The system measurement model will switch to be:
  (27) $$z=z_{i}^{pol}=H_{i}^{pol}x_{i}+v_{i}^{pol}$$

### 2.4. Multi-Mode Switching VBAKF Algorithm Summary

**Figure 2:**
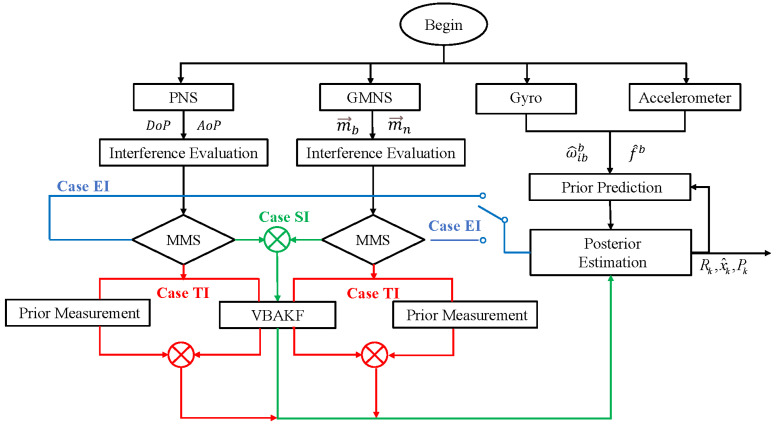
The flowchart of the MMS-VBAKF method.

To sum up, in the complex unfamiliar environment of pelagic seas, to achieve reliable navigation of ships, environmental uncertainty and multi-source interference need to be considered. Combining the variational Bayesian algorithm in Section 2.2 and the interference evaluation and multi-mode switching mechanism in Section 2.3, the flowchart of MMS-VBAKF method can be summarized in Figure 2.

When the measurement data are updated, the interference evaluation factors of the polarization system and the geomagnetic system are calculated separately. In the filter process, isolation processing is performed for the part of the system in this situation. For the situation where the measurement data contain non-negligible interference, the prior information and the measurement information are weighed. For the normal operating parts of the system, the variational Bayesian filter is used for adaptatively estimating the measurement statistics. In this way, by designing an interference evaluation algorithm and multi-mode switching mechanism, the environmental adaptability of the SINS/PNS/GMNS system in the uncertain practical application environment would be improved. In consequence, the MMS-VBAKF algorithm (Algorithm 1) proposed in this paper can be summarized as follows:
**Algorithm 1:** The MMS-VBAKF Algorithm**Input:**   When t=0, initialize:
   ${\hat{u}}_{0},{\hat{U}}_{0},R_{0},{\hat{c}}_{0},{\hat{C}}_{0},P_{0},Q_{0},{\hat{x}}_{0},\tau ,m,\rho ,d_{P},d_{R},Th_{pol}^{u},Th_{pol}^{d},Th_{mag}^{u},Th_{mag}^{d}$ **Navigation parameter estimation:**
   **For**
τ=1:L, do   (1) Obtain the polarization measurement values DoP and AoP, calculate   $\;\mathrm{the}\;\mathrm{polarization}\;\mathrm{mode}\;\mathrm{factor}\;\varsigma_{i}^{pol}$, and select the mode;   (2) Case SI: $\varsigma_{i}^{pol}=1$:      for i=0:N, do     The fixed-point iteration mechanism and the VBAKF algorithm are used to update ${\hat{u}}_{k}^{i+1},{\hat{U}}_{k}^{i+1},R_{k}^{i+1},{\hat{c}}_{k}^{i+1},{\hat{C}}_{k}^{i+1},P_{k|k-1}^{i+1},{\hat{x}}_{k}^{i+1},P_{k}^{i+1},K_{k}^{i+1}$ in real-time;     End for;  (3) Case TI: $\frac{Th_{pol}}{1-Th_{pol}}<\varsigma_{i}^{pol}<1$:     Update of the covariance matrix of polarization measurement noise:      ${\hat{R}}_{k}^{pol}=\left(1-\varsigma_{i}^{pol}\right)R_{k-1}^{pol}+\varsigma_{i}^{pol}{\tilde{R}}_{k}^{pol}$;     $\mathrm{Further}\;\mathrm{update}\;{\hat{c}}_{k},{\hat{C}}_{k},P_{k|k-1},{\hat{x}}_{k},P_{k},K_{k}$;   (4) Case EI: $\varsigma_{i}^{pol}=0$:      The polarization system was evaluated to be failed. Isolate the polarization system, and switch the system measurement model to:       $z=z_{i}^{mag}=H_{i}^{mag}x_{i}+v_{i}^{mag}$;     Restructure the integrated navigation system, and use fixed-point iteration mechanism and VBAKF algorithm to update ${\hat{u}}_{k}^{i+1},{\hat{U}}_{k}^{i+1},R_{k}^{i+1},{\hat{c}}_{k}^{i+1},{\hat{C}}_{k}^{i+1},P_{k|k-1}^{i+1},{\hat{x}}_{k}^{i+1},P_{k}^{i+1},K_{k}^{i+1}$ in real-time;  (5) Obtain the geomagnetic measurement value, calculate the geomagnetic mode switching factor, and select the mode;   (6) Case SI: $\xi_{i}^{mag}=1$:      for i=0:N, do     The fixed-point iteration mechanism and the VBAKF algorithm are used to update ${\hat{u}}_{k}^{i+1},{\hat{U}}_{k}^{i+1},R_{k}^{i+1},{\hat{c}}_{k}^{i+1},{\hat{C}}_{k}^{i+1},P_{k|k-1}^{i+1},{\hat{x}}_{k}^{i+1},P_{k}^{i+1},K_{k}^{i+1}$ in real-time;     End for;   (7) Case TI: $\alpha Th_{mag}^{d}<\gamma_{i}\leq \alpha Th_{mag}^{u}$:      The geomagnetic noise covariance matrix is updated to:      ${\hat{R}}_{k}^{mag}=\left(1-\xi_{i}^{mag}\right)R_{k-1}^{mag}+\xi_{i}^{mag}{\tilde{R}}_{k}^{mag}$;     Further update ${\hat{c}}_{k},{\hat{C}}_{k},P_{k|k-1},{\hat{x}}_{k},P_{k},K_{k}$;   (8) Case EI: $\xi_{i}^{mag}=0$:      It was evaluated that the geomagnetic system was out of work. Isolate the geomagnetic system, and switch the system measurement model to:          $z=z_{i}^{pol}=H_{i}^{pol}x_{i}+v_{i}^{pol}$;     Restructure the integrated navigation system, and use fixed-point iteration mechanism and VBAKF algorithm to update ${\hat{u}}_{k}^{i+1},{\hat{U}}_{k}^{i+1},R_{k}^{i+1},{\hat{c}}_{k}^{i+1},{\hat{C}}_{k}^{i+1},P_{k|k-1}^{i+1},{\hat{x}}_{k}^{i+1},P_{k}^{i+1},K_{k}^{i+1}$;   **End for** **Output:**
${\hat{x}}_{k},P_{k},R_{k},{\hat{u}}_{k},{\hat{U}}_{k},{\hat{c}}_{k},{\hat{C}}_{k}$


## 3. Results Analysis and Discussion

In order to verify the performance of the MMS-VBAKF algorithm, simulation experiments are carried out in this section. Considering that the interference of polarization measurement and geomagnetic measurement are multi-source, they have great uncertainty. In this part, the simulations are divided into three parts: random unknown noise, periodic sinusoidal noise, and part of the SINS/PNS/GMNS system fail.

### 3.1. Random Unknown Noise Situation

In this case, the integrated navigation system is working stably and the environmental conditions are well. Due to the uncertainty of the sensor’s perception from the external environment, the measurement noise is set as random and unknown. In the simulation, the initial time is set to 9:00 a.m. on 15 May 2021, the longitude is set to 120° E, the latitude is 39° N, and the altitude is 0 m. The date and position will be used for the calculation of the astronomical calendar and the world geomagnetic model.

The gyro drift bias of the inertial navigation system is 2.0 °/h and the random drift is 0.5 °/h. The accelerometer has a constant bias of 500 μg and a random bias of 50 μg. Both the data measurement frequency of the gyroscope and accelerometer is 10 Hz. Both the data measurement frequency of the polarization sensor and the geomagnetic sensor is 1 Hz. The filtering period of the integrated navigation is 1 s.

The noise settings for polarization measurement and geomagnetic measurement are as follows:

(28) $$v_{pol}∼N\left(0,\rho^{pol}\cdot \Sigma_{n}^{pol}\right)$$

(29) $$v_{mag}∼N\left(0,\rho^{mag}\cdot \Sigma_{n}^{mag}\right)$$

where $0\leq \rho^{pol}\leq 1$ is the coefficient related to the accuracy of the polarization sensor. $0\leq \rho^{mag}\leq 1$ is the coefficient related to the accuracy of the magnetic sensor. Both the polarization measurement noise covariance $\Sigma_{n}^{pol}$ and the geomagnetism measurement noise covariance $\Sigma_{n}^{mag}$ are time-varying values. The performance parameters of sensors in the simulation are summarized in Table 1.

**Figure 3:**
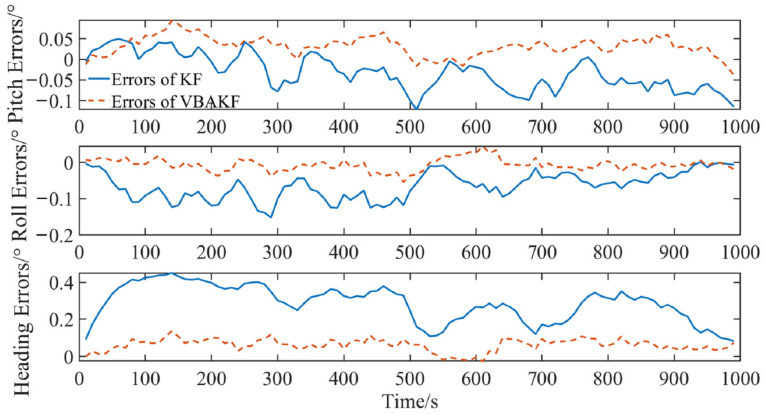
Comparison of filtering results when the measured noise covariance is an unknown time-varying random quantity.

**Figure 4:**
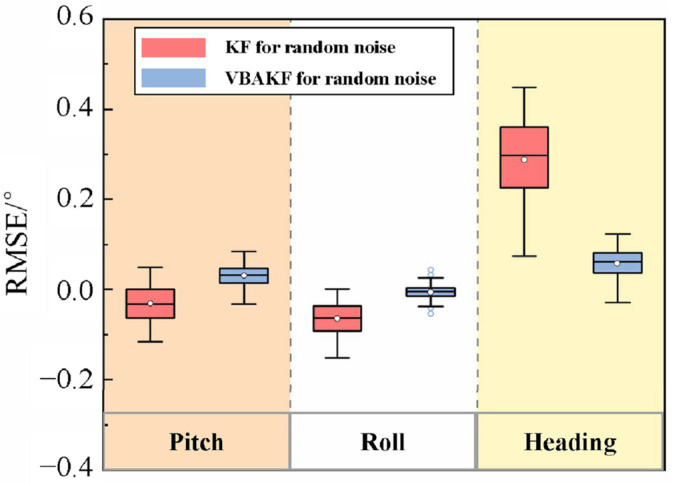
Filtering error box plot when the measured noise covariance is an unknown time-varying random quantity.

The simulation results are shown in Figure 3 and Figure 4.

It can be seen from Figure 3 and Figure 4 that with the random interference in the environment, the measurement noise covariance is no longer a constant value. For the measurement noises whose statistical characteristics change in real time, the Kalman filter results show greater fluctuation. By contrast, the VBAKF can estimate the noise covariance matrix in real time, and the filtering result is more stable. As can be seen in Table 2, the pitch angle estimation error RMSE calculated by the VBAKF algorithm is 0.038°, the roll angle estimation error RMSE is 0.017°, and the heading angle estimation error RMSE is 0.66°. Compared with KF, the accuracy is improved by 26.9%, 77.0%, and 78.2%, respectively.

### 3.2. Periodic Sinusoidal Characteristic Noise Situation

When the ship is sailing at sea, due to the influence of wind and waves, the hull will sway with the sea surface. It may cause periodic noises in the measured data of the integrated navigation system. Based on the previous section, in this case, we consider introducing noise of periodic sinusoidal characteristics besides random noise into the integrated navigation system.

The simulation conditions are set as the same in Section 3.2. The noise settings of the polarization measurement and geomagnetic measurement are as follows:

(30) $$v∼\rho^{pol}\cdot N\left(0,\Sigma_{n}^{pol}\right)+0.8\sigma \cdot \sin(\frac{\pi \cdot i}{27}),i=1,2,3\ldots$$

(31) $$v∼\rho^{mag}\cdot N\left(0,\Sigma_{n}^{mag}\right)+0.8\sigma \cdot \cos(\frac{\pi \cdot i}{21}),i=1,2,3\ldots$$

where σ represents the degree of wind and waves on the sea surface and i is the sensor measurement update sequence.

**Figure 5:**
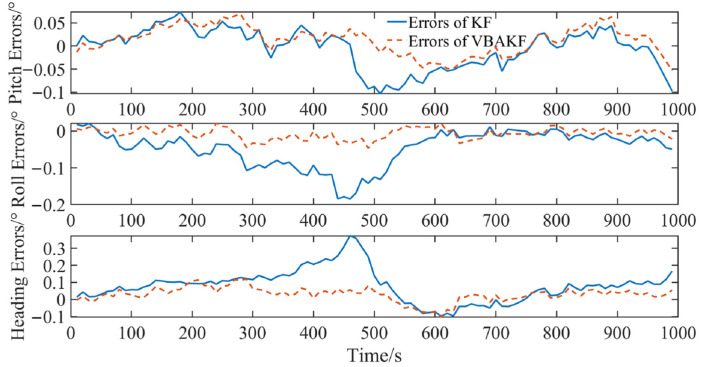
Comparison of filtering results when the measured noise is a random quantity with periodic sinusoidal characteristics.

**Figure 6:**
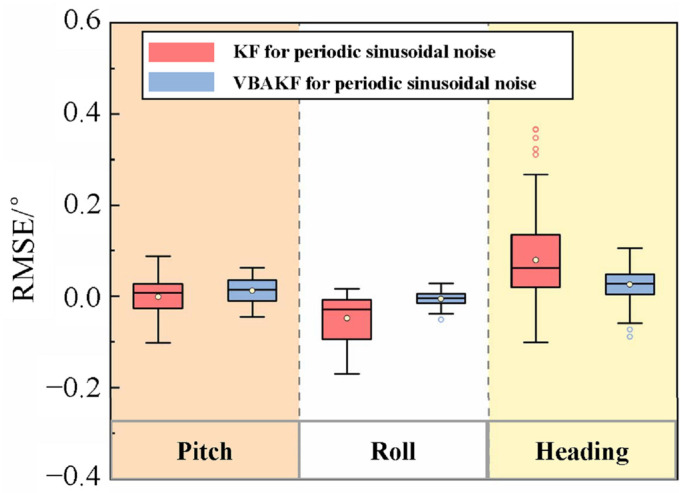
Filtering error box plot when the measured noise covariance is a random quantity with periodic sinusoidal characteristics.

We perform the simulation under the above conditions, and the results are shown in Figure 5 and Figure 6.

It can be seen from Figure 5 and Figure 6 that when the sea surface waves cause noise with periodic sinusoidal characteristics in the sensor measurement data, the filtering results also show certain fluctuation. Compared with the KF algorithm, the proposed VBAKF algorithm manifests better anti-sway characteristics. It is especially obvious in the time period of 400 s to 500 s. According to the analysis of the noise statistical characteristics in Figure 6 and Table 3, the estimated error RMSE of the pitch angle, roll angle, and heading angle of the VBAKF algorithm are 0.029°, 0.015°, and 0.046°, respectively. Compared with the KF algorithm, the results are improved by 29.2%, 78.8%, and 64.1%, respectively.

### 3.3. Situation Where Part of the Integrated Navigation System Become Invalid

In this section, extreme environments of the pelagic sea are considered. For the ship performing missions, the harsh environmental conditions may cause serious interference in the integrated navigation system. In this situation, to verify the validity of the interference detection and evaluation algorithm and the corresponding mode switching algorithm, a set of simulations are carried out. To be specific, in the process of generating polarization data and geomagnetic data, we set the case of polarization sensor shielding interval and magnetic interference interval as shown in Table 4.

**Figure 7:**
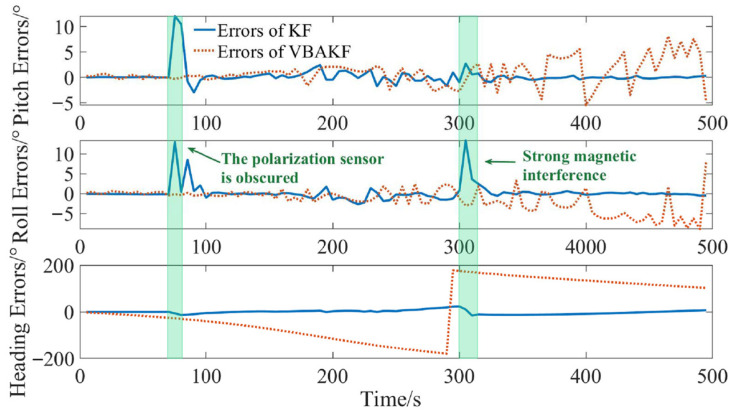
The three-dimensional attitude estimation results with the serious interference cases.

Without coping with the interference evaluation and isolation, the filtering results are shown in Figure 7.

**Figure 8:**
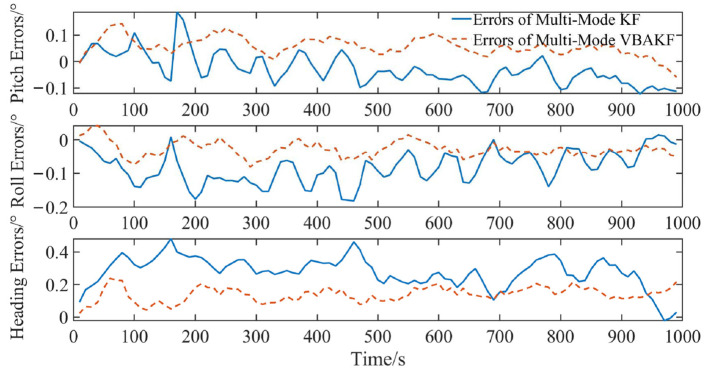
Comparison of filtering results with interference evaluation and multi-mode switching algorithms.

**Figure 9:**
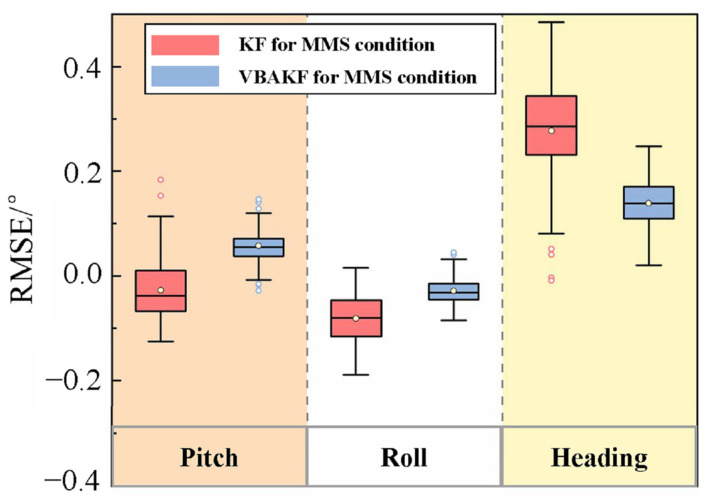
Filter error box plot of interference evaluation and multi-mode switching algorithm.

It can be seen from Figure 7 that in the time interval (0 s, 140 s), the integrated navigation system can provide the ship with an accurate pitch angle, roll angle, and heading angle reference. In the time interval (140 s, 160 s), due to the shelter in polarization sensor, the polarization measurement data contain a large error, and the estimation result of the three-dimensional attitudes showed errors greater than 10°. In the time interval (160 s, 600 s), even if no polarization or magnetic interference is applied, the accuracy estimated of three-dimensional attitudes became very poor. When magnetic interference occurs in 600 s, the heading estimation result even showed an error of more than 20°. Therefore, from the above results, interference evaluation and isolation must be considered. Adopting the interference evaluation and the mode switching mechanism proposed in the paper, the results are shown in Figure 8 and Figure 9.

It can be seen from Figure 8 that the proposed interference evaluation and multi-mode switching mechanism took effect. In the time interval (140 s, 160 s) when the polarization system failed and the time interval (600 s, 630 s) when the geomagnetic system failed, the integrated navigation system did not experience degradation of accuracy, and the mode switching was effectively completed. In addition, from Figure 9 and Table 5, the filter estimation error RMSEs of the pitch angle, roll angle, and heading angle of the multi-mode switching KF algorithm are 0.066°, 0.093°, and 0.293°, respectively. The error RMSEs of the multi-mode VBAKF algorithm are 0.067°, 0.035°, and 0.147°, respectively. The accuracy of roll angle and heading angle has been significantly improved by 40.8% and 49.8%, respectively.

## 4. Conclusions

The SINS/PNS/GMNS integrated navigation system can play a role in meeting the ship’s requirements of reliable autonomous navigation in the unfamiliar pelagic sea. However, the precision of obtained data of PNS and GMNS relies on the external environment, in which there exist many uncertain and unknown interferences. To solve this problem, the interference evaluation strategy is designed and the MMS-VBAKF algorithm is proposed. By the strategy and algorithm, the interference that the SINS/PNS/GMNS integrated navigation system suffers is divided into three cases to be dealt with. For case SI, the unknown statistical properties of the measurement noise and the system states can be estimated adaptively by the VBAKF. For case TI, the evaluation factors are used to weigh the measurement information at the moment and in the previous time. In this way, the stability and robustness of the SINS/PNS/GMNS integrated navigation are improved. For case EI, as the system suffers serious interference, the multi-mode switching mechanism is performed at first, then the navigation states can be estimated reposefully. The method proposed in this paper has a certain reference significance for the exploration of ship autonomous navigation technology in the unfamiliar pelagic sea.

## Figures and Tables

**Table 1:** The performance parameters of sensors in the simulation.

Sensors	Performance Parameters	Frequency
Gyro	constant drift: 2.0 °/h random drift: 0.5 °/h	10 Hz
Accelerometer	constant bias: 500 μg random bias: 50 μg	10 Hz
Polarization sensor	$N\left(0,\rho^{pol}\cdot \Sigma_{n}^{pol}\right)$	1 Hz
Geomagnetic sensor	$N\left(0,\rho^{mag}\cdot \Sigma_{n}^{mag}\right)$	1 Hz

**Table 2:** The RMSE result of the three-dimensional attitude estimation errors when the measured noise covariance is an unknown time-varying random quantity.

Method	RMSE (/°)		
	Pitch	Roll	Heading
KF	0.052	0.074	0.303
VBAKF	0.038	0.017	0.066

**Table 3:** The RMSE result of the three-dimensional attitude estimation errors when the measured noise covariance is a random quantity with periodic sinusoidal characteristics.

Method	RMSE (/°)		
	Pitch	Roll	Heading
KF	0.041	0.071	0.128
VBAKF	0.029	0.015	0.046

**Table 4:** The simulation settings of interference cases in the navigation process.

	Time Interval	Whether to Interfere	Cause of Interference
**Case 1**	(0 s, 140 s]	no	/
**Case 2**	(140 s, 160 s]	Polarization interference	Sensor occlusion, etc.
**Case 3**	(160 s, 600 s]	no	/
**Case 4**	(600 s, 630 s]	Magnetic interference	Submarine iron–nickel ore, etc.
**Case 5**	(630 s, 1000 s]	no	/

**Table 5:** The RMSE result of the three-dimensional attitude estimation errors of the interference evaluation and multi-mode switching algorithm.

Method	RMSE (/°)		
	Pitch	Roll	Heading
KF	0.066	0.093	0.293
VBAKF	0.067	0.035	0.147

## Data Availability

Not applicable.
